# Notes and News

**Published:** 1897-10-09

**Authors:** 


### Notes and News.

(Collected and Communicated.)

Mrs. Cleaver has presented a portrait of her late husband, Dr. Cleaver, to the Cleaver Ward of the Sheffield Children's Hospital. The ward was erected to the memory of Dr. Cleaver by his friends, and opened last year.

The new convalescent home at Skegness has just been opened by Mr. Hooley, who has largely contributed to the scheme. The home consists of four new dwelling-houses, situated in the Grosvenor Road, and having a south aspect. The houses have been adapted for the purposes of a home, the ground floor rooms being thrown into one. Twenty-eight bed-rooms are provided, two bath-rooms, and offices. The decorations are handsome.

Clinical lectures or demonstrations (free to students and practitioners) will be delivered at the National Hospital for Paralysis, Queen's Square, at half-past three on the following dates: October 12th, Dr. Buzzard; 19th, Dr. Ferrier, F.R.S.; 26th, Dr. Ormerod; November 2nd, Dr. Beevor (on the examination of the action of muscles); 9th, Dr. Tooth (on spinal cord lesions, with lantern demonstration); 16th, Sir F. Semon (on the diagnostic significance of laryngeal abductor paralysis); 23rd, Mr. Marcus Gunn (on paralysis of the ocular muscles); 30th, Mr. Victor Horsley, F.R.S. (surgery of the nervous system); December 7th, Mr. Ballance (surgery of the nervous system); 14th, Dr. Colman; 21st, Dr. Taylor.

The medical school authorities at the Middlesex Hospital have decided to enlarge and reconstruct their school buildings. The museum and the large lecture-hall will remain untouched. The new block is to contain on the ground floor a complete chemical department with a chemical laboratory, private laboratory, lecture hall, and lecturers'-room. On the upper floor the pathological department will be situated, and the department will be in direct communication with the waiting-room. Above this again will be the anatomical class and the dissecting-rooms. The basement and ground floor of the other new block will be occupied by the extensive physiological laboratory and its various offices. The buildings are to be completed before the end of the year.

St. Mary's Hospital, Paddington, which has been closed for two months for extensive repairs, has now been reopened for the reception of in-patients, and the out-patients' department has also resumed work.

The convalescent home of St. George's Hospital at Wimbledon is at present closed for redrainage, and this opportunity is being taken to do some necessary cleaning and repairs.

H.R.H. the Duke of Cambridge, K.G., will visit Bath on October 18th for the purpose of opening the Grand Pump Room Annexe and Roman Promenade, now in course of completion at a cost of nearly £30,000, and attached to the hot mineral springs.

The Cardiff Infirmary has been attacked on the subject of extravagant expenditure, but it has been pointed out that the accusation is unfounded, seeing that the cost per bed at Cardiff is only £54 1s. 4½d., whilst at 34 similar institutions in the country it averages upwards of £64.

Added to the other dangers of an expedition to the Klondyke Goldfields, we now learn that typhoid fever has broken out at Dawson City. The large sums reported to be paid for medical attendance are enough to tempt the needy practitioner to contemplate trying his fortune in such a veritable goldfield.

The haircutters in Paris have been placed under regulations formulated by the Department of Hygiene. All their appliances are to be inspected and chosen from a hygienic point of view, and distinct rules are laid down for their procedure generally in carrying out their calling.

Butler, in the United States, is to have a general hospital. Nearly 10,000 dollars has been subscribed. At Asheville, Mr. George Vanderbilt is erecting a hospital for tuberculosis. Mr. Vanderbilt is determined that this hospital, which he will build and maintain himself, shall be second to none of its kind.

A good deal of interest is created by the annual inspection of the fever hospitals in Liverpool by the Port Sanitary and Hospitals Committee. This annual "lime-light" of inspection is an excellent thing, because it not only infuses life and interest into institutions necessarily removed from participation in visitations by the public, but it gives an opportunity for wholesome competition and rivalry in efficiency. The visitors pass from one institution after the other, and the expert amongst them are glad of opportunities of contrast and comparison to relieve what might otherwise be a monotonous procession.

The Duke of Cambridge visited York last week for the purpose of laying the foundation-stone of the additional premises to be added to the County Hospital. A portion of the Jubilee offerings of the city of York is to be devoted to the erection of a new ward, to be called the Victoria Ward, to be situated in the new wing. This wing is to consist of a children's ward for 18 cots, two observation wards with two beds in each, a day-room, ward kitchen and offices, and detached lavatory blocks. At the south end there will be a glazed verandah. The building is to be fireproof throughout the walls lined with tiles, and the flooring of teak.

